# Molecular characterization of *Vitellogenin-*like1 gene in *Sogatella furcifera* (Hemiptera: Delphacidae), and its function on reproduction

**DOI:** 10.1093/jisesa/ieae013

**Published:** 2024-02-27

**Authors:** Changyu Xu, Fei Li, Maolin Hou, Yudi Liu

**Affiliations:** State Key Laboratory for Biology of Plant Diseases and Insect Pests, Institute of Plant Protection, Chinese Academy of Agricultural Sciences, Beijing, PR China; State Key Laboratory for Biology of Plant Diseases and Insect Pests, Institute of Plant Protection, Chinese Academy of Agricultural Sciences, Beijing, PR China; State Key Laboratory for Biology of Plant Diseases and Insect Pests, Institute of Plant Protection, Chinese Academy of Agricultural Sciences, Beijing, PR China; State Key Laboratory for Biology of Plant Diseases and Insect Pests, Institute of Plant Protection, Chinese Academy of Agricultural Sciences, Beijing, PR China

**Keywords:** *Sogatella furcifera*, vitellogenin-like1, RNAi, ovary, testis

## Abstract

In this study, a vitellogenin like1 gene (*SfVg-*like1) in *Sogatella furcifera* was identified. The open reading frame (ORF) encoded 1,321 amino acid sequence. Structure analysis reveals that the amino acid sequence of *SfVg-*like1 has 3 conserved LPD_N, DUF1943 and VWFD domains. Phylogenetic analyses showed that *SfVg-*like1 was clustered in the same branch with the *Vg-*like1 of *Nilaparvata lugens* (100% bootstrap value) compared with other Hemiptera insects *Vg*s associated with vitellogenesis. Temporo-spatial expression analyses showed that *SfVg*-like1 expressed during all stages, and in both genders. The relative expression levels of *SfVg*-like1 mRNA were higher in adults than in nymph developmental stages. The knockdown of *SfVg-*like1 gene resulted in the inhibition of the ovarian development in female adults, whereas the morphology of the testis in male adults was not been affected. The silence of *SfVg-*like1 could decrease the relative expression levels of target of rapamycin (*SfTOR*, GenBank MW193765) and vitellogenin (*SfVg*, GenBank MH271114) genes significantly in female adults. However, the knockdown of *SfTOR* or *SfVg* genes in female adults did not affect the transcript level of *SfVg-*like1. Therefore, it demonstrated that *SfVg-*like1 might locate on the upstream signaling pathways of *SfTOR* and *SfVg*. These results demonstrate that *SfVg-*like1 is essential for *S. furcifera* reproduction, and it could be the potential target for the control of this pest.

## Introduction

The white-backed planthopper (WBPH) *Sogatella furcifera* (Horváth) (Hemiptera: Delphacidae) is one of the most important rice pests in Asian countries. It is a phloem-feeding hemipteran insect and can cause serious damage by sucking rice sap and transmitting *Southern rice black-streaked dwarf virus*. Furthermore, WBPH has rapid reproduction rate and egg production, and each female adult can produce more than 400 offspring throughout the lifetime of them under appropriate conditions (Cheng 1996). The vitellogenin (*Vg*) and vitellogenin-related (*Vg*-like) genes play important role during the oogenesis biosynthesis and its accumulation in oocytes (Raikhel and Dhadialla 1992, Li et al. 2020).

Due to the various gene duplication events, a diverse class of *Vg* genes has emerged in many insects over time, termed the *Vg*-like genes, which are thought to be associated with insect reproduction, adaptation, and evolution (Lynch and Conery 2000, Garcia et al. 2010, Richards 2019). For example, the white perch (*Morone americana*) has 3 types of Vg proteins: VtgAa, VtgAb, and VtgC, which appear to be associated with different functions during vitellogenic oocyte growth (Schilling et al. 2015). Three *Vg* homologs, Vg-likeA, Vg-likeB, and Vg-likeC, have emerged in Hymenoptera, which are orthologous to the conventional *Vg*s but with different conserved protein domains (Morandin et al. 2014). The study of reproduction-related genes in 3 fruit fly species showed that *Zeugodacus cucurbitae* (Coquillett), *Bactrocera dorsalis* (Hendel), and *Ceratitis capitata* (Wiedemann) had 7, 11, and 1 vitellogenin-related genes, respectively (Wu et al. 2022). Chen et al. (2022) identified 4 *Vg* genes in *Z. cucurbitae*, and knockdown of them can delay the ovarian developments of female adults. Two *Vg*-like genes were reported in the brown planthopper, *Nilaparvata lugens*, and phylogenetic analyses showed that they are not clustered with the conventional insect *Vg*s associated with vitellogenesis, and knockdown of them in female adults resulted in failure to egg hatch or death (Shen et al. 2019).

At present, several oviposition-related genes have been reported in WBPH, such as target of rapamycin (*TOR*) (GenBank MW193765), *Vg* (GenBank MH271114, MN229743, and MN296092), vitellogenin receptor (*VgR*) (GenBank MN327568 and MN296093), and *Vg*-like (GenBank MN296091) (Hu et al. 2019b, Zhou et al. 2020, Yi et al. 2021). The genes of *TOR*, 3 *Vg*, and 2 *VgR* have been identified to play important roles on oocyte maturation of WBPH female adults (Hu et al. 2019b, Zhou et al. 2020, Yi et al. 2021). However, the biological and molecular functions of *Vg*-like gene in WBPH remain largely unclear and need further to investigate.

In this study, we cloned the WBPH *Vg-*like1 (*SfVg-*like1) gene. Its basic molecular and structural characteristics, and the phylogenetic relationship with other reported insect *Vg* related genes were investigated. In addition, the dsRNA-mediated gene knockdown method was employed to analyze the effect of *SfVg-*like1 on the developments of the WBPH ovary and testis. Finally, the interaction effects were investigated to understand the regulation mechanism among *SfVg-*like1, *SfVg* (GenBank MH271114) and *SfTOR* using RNAi technique. The functional research of *SfVg-*like1 gene will give aid for providing it as the potential target of RNAi-based WBPH control.

## Materials and Methods

### Insects

WBPH colonies were collected from rice fields in Xing’an County (Latitude 25.61°N, Longitude 110.67°E), Guilin City, Guangxi Zhuang Autonomous Region, China. More than 5 generations were reared on Taichuang Native 1 (TN1) rice seedlings in a controlled climate room that was maintained at 27 ± 1°C, 75 ± 10% relative humidity, and L:D = 16:8.

### Cloning the Full Length of *SfVg*-like1 cDNA

The rapid amplification of cDNA ends polymerase chain reaction (RACE-PCR) method was used to clone the full-length cDNAs of *SfVg*-like1. To obtain the full-length *SfVg*-like1 cDNA, gene-specific primers (Table 1) were designed based on the partial cDNA of *SfVg*-like1 that was extracted from the whole WBPHs and synthesized for 3ʹ- and 5ʹ- RACE using a SMARTer^™^ RACE cDNA Amplification Kit (Clontech, Mountain View, CA, USA) following the recommendations given by the manufacturer. The PCRs were executed under the following sequential conditions: incubation at 94°C for 3 min, followed by 30 cycles at 94°C for 30 s, 65°C for 30 s, and 72°C for 2 min. The PCR products were cloned into the pEASY-T5 zero vector (TransGen, Beijing, China) and sequenced.

**Table 1.** The primers used in this study.

Primers	Primer sequence (5ʹ-3ʹ)
**Race**	
*3´GSP*	gattacgccaagcttCAGTTGGATTGCGGAGTCGGAAGGG
*3´Nested GSP*	gattacgccaagcttGGAGGAAGACGGCGTTCAGACCTTTC
**qPCR**	
q*SfVg*-like1-F	AGCAGCCTCTCATCCAAATC
q*SfVg*-like1-R	GACTGCCATCCTTCTCCATTAC
*L9-F*	CAAGATGAGAGCCGTGTA
*L9-R*	CGAGTTGGTAACAGTGAC
*L10-F*	GCGACTTCATCCGTTCCA
*L10-R*	CACTCTAGCCACTGTTCCTT
**RNAi**	
ds*Vg*-like1-T7-F	taatacgactcactatagggGAGAGCGCTGAAGAAACAAATC
ds*Vg*-like1-T7-R	taatacgactcactatagggACAAGTAGCGGCCAGAATATC
ds*GFP*-T7-F	TAATACGACTCACTATAGGGGGAGAAGAACTTTTCACTGG
ds*GFP*-T7-R	TAATACGACTCACTATAGGGAGTTGAACGGATCCATCTTC

### Bioinformatic and Phylogenetic Analyses

The cloned sequence of *SfVg*-like1 was identified using the online BLAST program on the National Center for Biotechnology Information (NCBI) website (http://www.ncbi.Nlm.Nih.Gov/BLAST/). The isoelectric point and molecular weight of *SfVg*-like1 were calculated using ExPASy online software (http://web.expasy.org/compute_pi/). The signal peptides were predicted by Signal IP software (http://www.cbs.dtu.dk/services/SignalP/) and the conserved domains were analyzed on SMART website (https://smart.embl-heidelberg.de/).

Amino acid sequences of *SfVg*-like1 from 20 other insect species, 6 WBPH genes including 1 *Vg*-like, 2 *VgR*s and 3 *Vg*s were downloaded from the GenBank database, and the detailed information about the names and GenBank numbers of these genes were listed in Supplementary Table S1. The Clustalx program (Thompson et al. 1997) was used to align the amino acid sequences. A neighbor-joining (NJ) phylogenetic tree was constructed using on a poisson model and MEGA 6.0 software with 1,000 bootstrap replications (Tamura et al. 2013).

### Developmental Expression Profiles of *SfVg*-like1

Total RNA was isolated from whole insects at various developmental stages using RNAiso Plus kit (TaKaRa, Tokyo, Japan) according to the manufacturer’s recommendations. Samples were collected from third-instar nymphs (every 24 h after molting, *n *= 40), fourth-instar nymphs (every 24 h after molting, *n *= 20), fifth-instar nymphs (every 24 h after molting, *n *= 20), female adults (every 12 h after molting, *n *= 10), and male adults (every 12 h after molting, *n *= 10). The concentration of total RNA was measured using a NanoDrop 2000 spectrophotometer (Thermo Fisher Scientific, Bremen, Germany), and 1,000 ng RNA was used as a template to synthesize the first-strand cDNA using a FastKing RT Kit (Tiangen, Beijing, China) according to the manufacturer’s protocol. Then, the cDNA samples were immediately frozen at –80°C and stored for further experiments.

To investigate the developmental patterns of *SfVg*-like1, real-time qPCR was conducted on an ABI 7500 Real-Time PCR System (Applied Biosystems, Carlsbad, CA, USA) using gene-specific primer pairs designed by Primer Premier 5 (Table 1).

The total volume of 20 µl for each reaction contained 10 µl of SYBR Premix Ex TaqⅡ (TaKaRa, Tokyo, Japan), 0.4 µl of 50 × ROX Reference Dye II, 4 µl of cDNA (16 ng), 0.4 µl (10 µM) each primer, and 4.8 µl of distilled water, and conducted under the following sequential conditions: initial denaturation at 95°C for 30 s, followed by 40 cycles at 95°C for 5 s and 60°C for 34 s. Using WBPH ribosomal protein L9 (GenBank KP735523) and L10 (GenBank KP735524) as reference genes (An et al. 2016), the relative real-time quantitative PCR (RT-qPCR) data were calculated with the delta CT method (Livak and Schmittgen 2001).

### RNA Interference

The functions of *SfVg-*like1 gene were investigated using RNA interference method with the ds*GFP* as a parallel control. For dsRNA preparation, the *SfVg-*like1 was first amplified using gene-specific primers (Table 1) conjugated with the T7 polymerase promoter sequence. The gel purified PCR products were used as templates to synthesize dsRNA (The length of *SfVg-*like1 was 802 bp) using the HiScribe T7 Quick High Yield RNA Synthesis Kit (New England Biolabs, Ipswich, MA, USA) according to the manufacturer’s protocol. The quality and size of the dsRNA products were checked by 1.5% agarose gel electrophoresis, and then diluted with the diethyl pyrocarbonate-treated water to a final concentration of 5.0 µg/µl and stored at –80°C until use (Supplementary Fig. S1).

RNAi technique was performed according to our previous method. Briefly, WBPH female and male adults newly emerged within 24 h of emergence were used for microinjections. Before dsRNA injection, the WBPH was anesthetized with carbon dioxide for 10 s, and then each WBPH was injected with 200 ng dsRNA into the conjunction prothorax and mesothorax using a FemtoJet (Eppendorf-Netheler-Hinz, Hamburg, Germany). Post-injection, the female and male adults were reared on 5-6-leaf-stage rice seedlings for 1, 3, 5, and 6 d for different treatment. The injected 40 female and 40 male adults were selected for each treatment, with 3 replicates in each group.

RT-PCR was used to evaluate the efficiency of RNA silence at 1, 3, and 5 d after injection. The female ovaries and male testes at day 3 and 6 after dsRNA injection were dissected using micro forceps (Shanghai Medical Instruments Ltd., Corp., Shanghai, China) to validate the *SfVg*-like1 knockdown influences on their developments. Before dissection, the WBPHs were anesthetized on ice, and the tissues were removed using forceps and placed in 1 × phosphate buffered solution. The phenotypes of the ovaries and testes were observed and photographed using an Olympus stereomicroscope (SZX16, Olympus, Tokyo, Japan).

### Statistical Analyses

All the results were based on the mean values excluding the morphology figure. For the expression levels of *SfVg*-like1 at different developmental stages, the significant differences were checked through one-way analysis of variance, followed by least-significant difference test for 2 sample comparisons and Tukey’s test for multiple comparisons. Student’s *t*-test was used to compare 2 treatments for gene knockdown on expression levels of *SfVg*-like1, *SfVg* and *SfTOR* (*P < 0.05, **P < 0.01). The error bars represent the means ± standard errors. All analyses were conducted in IBM SPSS Statistics version 22.0 (SPSS Inc., Chicago, IL, USA).

## Results

### Sequence and Structure of *SfVg*-like1

The cDNA sequence of *SfVg*-like1 was identified by cloning and sequencing (4,222 bp, GenBank: OP882573). The open reading frame (ORF) of 3,966 bp encoded 1,321 amino acid sequence with a theoretical isoelectric point (pI) of 6.22 and a molecular mass of 147.49 kDa. The 18 amino acids signal peptide sequence (MDRFVIIVLGLLIAGSAS) is at N-terminus (Supplementary Fig. S2). Analysis of the *SfVg*-like1 amino acid sequence by InterPro Scan search predicted 3 conserved regions: Lipoprotein N-terminal (LPD_N, amino acids 25–579), the unknown function motif 1943 (DUF1943, amino acids 610–873) and von Willebrand factor type D domain (VWD domain, amino acids 1,161–1,321) (Supplementary Fig. S3).

**Fig. 1.**
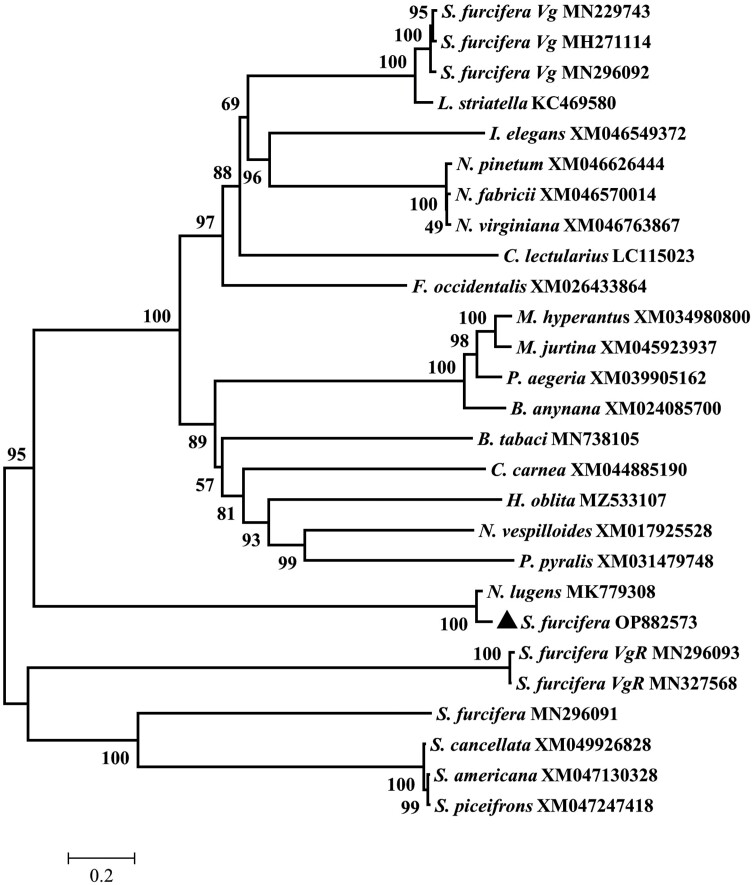
Phylogenetic tree of *SfVg*-like1 constructed using the neighbor-joining method. Bootstrap values (1,000 replications) indicated at each node. The black triangle stands for amino acid sequence of *SfVg*-like1.

According to the BLAST search results in NCBI database, *SfVg*-like1 shares 83.93% similarity with *N. lugens Vg*-like1 gene (GenBank: MK779308). Using the maximum likelihood method, *SfVg*-like1 was compared phylogenetically with other 20 insect *Vg*-like genes, 1 *Vg*-like, 2 *VgR*, and 3 *Vg* genes of WBPH based on amino acid sequences. In the phylogenetic tree, *SfVg*-like1 was clustered on the same branch with the *Vg*-like1 of *N. lugens* (100% bootstrap support value) (Fig. 1), whereas it was divided into different groups with *Vg*, *Vg*-like and *VgR* of WBPH.

### *SfVg*-like1 Expression Profiles

**Fig. 2.**
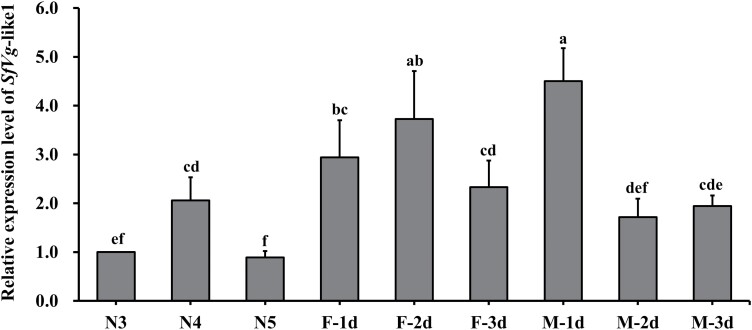
Expression patterns of *SfVg*-like1 at different developmental stages. N3, N4, and N5: third, fourth, and fifth nymphal instar; F (M) -1d, -2d, and -3d: female (male) adults at day 1, 2, and 3 after emergence. The different lowercase letters above the columns indicate significant differences (Tukey test, *P *< 0.05). Bars, ± SEM. SEM, standard error of the mean.

We determined the relative expression of *SfVg*-like1 in nymphs (third to fifth instar) and female and male adults emerged within 1–3 days using qRT-PCR. *SfVg*-like1 was expressed in all tested development stages, and the expression levels in adults were higher than the nymph stage. *SfVg*-like1 transcripts were detected in both females and males, with high expression level that peaked in newly emerged adults at the first day of male, and female adult stage showed higher expression level than male adult stage at the second and third day (Fig. 2).

### Effects of RNAi Knockdown of *SfVg*-like1

**Fig. 3.**
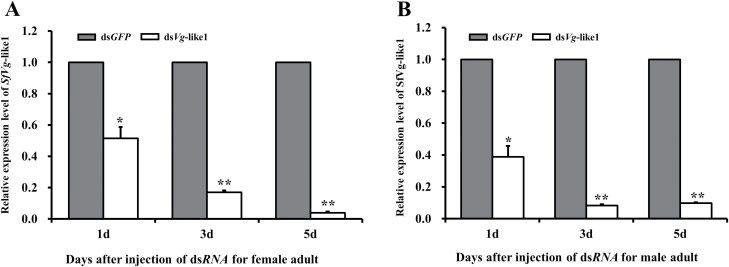
Relative expression levels of *SfVg*-like1 at day 1, 3, and 5 for A) female and B) male adults after ds*Vg*-like1 injection. Ds*GFP*:control group of *S. furcifera* injected with *GFP* dsRNA; ds*Vg*-like1: *S. furcifera* treated with *Vg*-like1 dsRNA. *P < 0.05, **P < 0.01 (Student’s *t-*test). Bars ± SEM.

As the expression of *SfVg*-like1 in nymphs was at low level, *SfVg*-like1 RNAi was only conducted on female and male adults within 24 h of emergence. *SfVg*-like1 mRNA levels were measured at day 1, 3, and 5 post-injection. RNAi efficiently downregulated their expression levels in female adults by 48.5% (1 d), 83% (3 d), and 96.1% (5 d), and in male adults by 61.1% (1 d), 91.7% (3 d), and 90.2% (5 d), respectively, compared with those of the ds*GFP*-treated control (Fig. 3A and B).

**Fig. 4.**
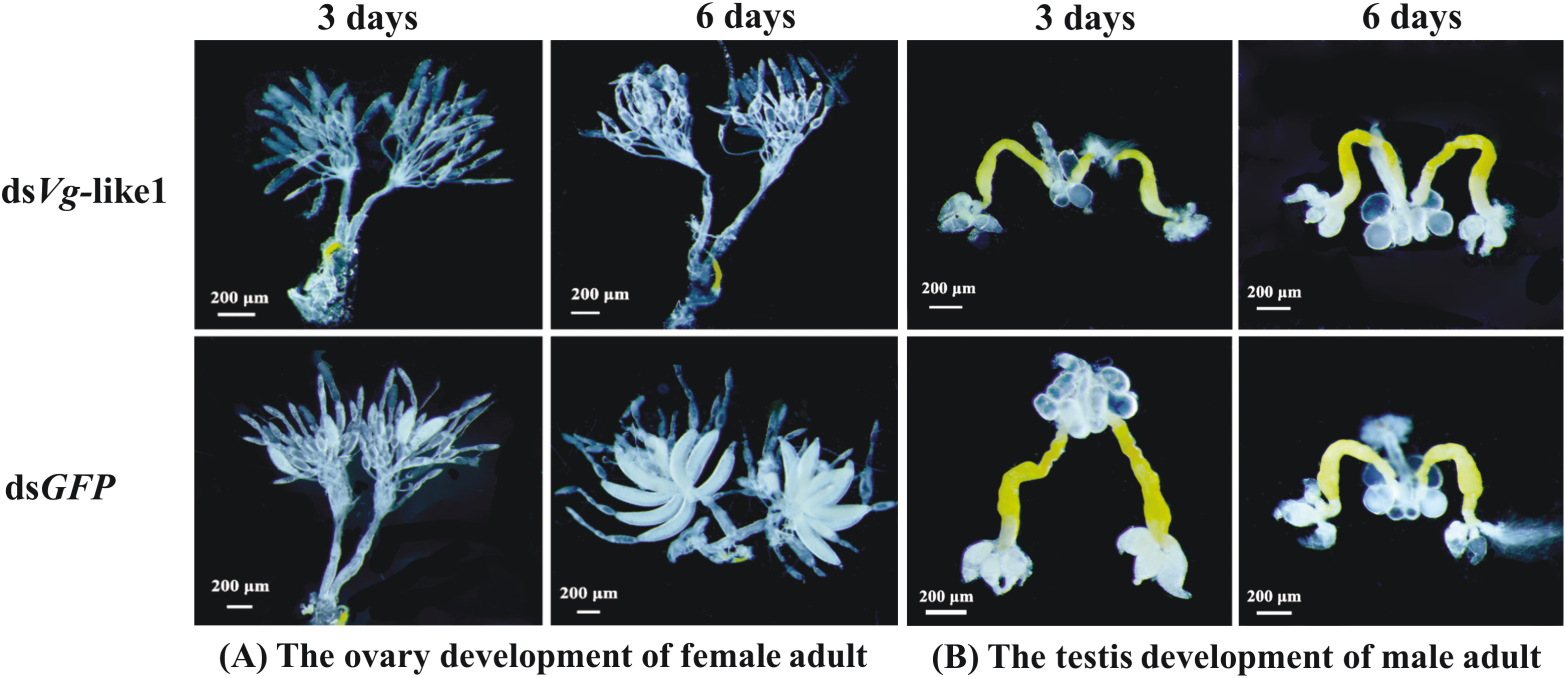
Effects of *SfVg*-like1 silencing on the development of the ovaries and testes at day 3 and 6 for adults after ds*Vg*-like1 injection within 24 h of emergence. A) Female and B) male adults injected with ds*Vg*-like1.

To determine the effect of RNAi knockdown on the reproductive development of adults, we dissected the female ovaries and male testes at day 3 and 6 after dsRNA injection. *SfVg*-like1 knockdown severely inhibited the oocyte growth in the ovarioles, and caused the misshapen and irregular edges oocytes (Fig. 4A). This malformation indicated that *SfVg*-like1 was required for oogenesis and oocyte maturation, and nearly all ds*SfVg*-like1 treated females failed to produce eggs. However, no obvious testis malformations were observed in males at day 3 and 6 after dsRNA injection (Fig. 4B). The above results indicated that *SfVg*-like1 silencing could effectively inhibit the female ovarian development but had no significant effect on male testes development.

### Effects of RNAi Knockdown on the Expression of SfVg-like1, SfVg, and SfTOR

**Fig. 5.**
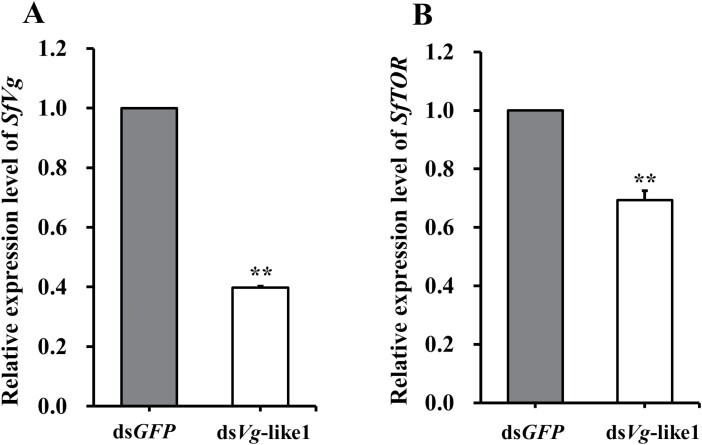
Relative expression levels of A) *SfVg* and B) *SfTOR* for *S. furcifera* female adults at day 5 after ds*Vg*-like1 injection. Ds*GFP*: control group of *S. furcifera* injected with *GFP* dsRNA; ds*Vg*-like1: *S. furcifera* treated with *Vg*-like1 dsRNA. **P < 0.01 (Student’s *t-*test). Bars ± SEM.

**Fig. 6.**
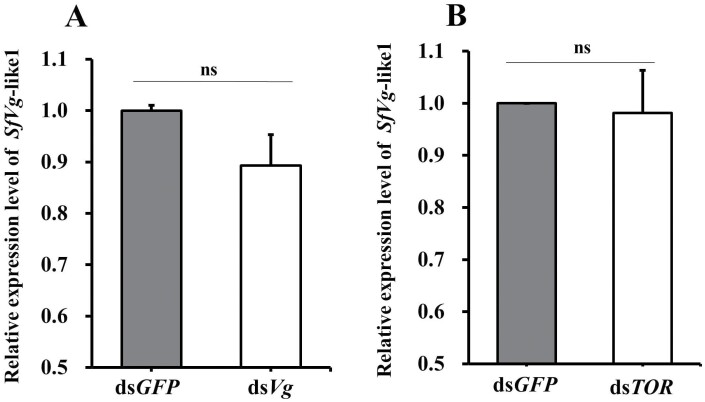
Relative expression levels of *SfVg*-like1 in *S. furcifera* female adults at day 5 after injection of ds*Vg* (A) and ds*TOR* (B). Ds*GFP*: control group of *S. furcifera* injected with *GFP* dsRNA; ds*Vg*/*TOR*: *S. furcifera* treated with *Vg*/*TOR* dsRNA. Ns: no significant difference (Student’s *t-*test). Bars, ± SEM.

The female adults were injected with ds*RNA* of *SfVg*-like1, *SfVg*, or *SfTOR* within 24 h of emergence, and the expression levels of them were detected at day 5 post-injection. The qRT-PCR results showed that *SfVg*-like1 silencing influenced the transcript levels of both *SfVg* and *SfTOR* of female adults, and the expression levels of them were 60.2% and 37.1% lower, respectively, than those of the ds*GFP*-treated controls (Fig. 5A and B). However, injection of either ds*Vg* or ds*TOR* had no significant effect on the transcript levels of *SfVg*-like1 (Fig. 6A and B).

## Discussion

Insects harbor more than 1 *Vg*-like genes, and these genes differ in their expression patterns, mode of evolution, and number of conserved protein domains (Morandin et al. 2014). In this study, we identified the coding sequence of a WBPH *Vg*-like gene named as *SfVg*-like1. The amino acid sequence of SfVg-like1 ORF showed 97.66% similarities with that of *N. lugens* Vg*-*like1 ORF, whereas it had only 48.44% similarities with the ORF amino acid sequence of the reported WBPH *Vg*-like. The phylogenetic tree analysis based on amino acid sequences revealed that *SfVg*-like1 shared a close phylogenetic relationship with the *N. lugens Vg*-like1 gene, whereas was distant from the *Vg*-like, 3 *Vg*, 2 *VgR* genes of WBPH. Hence, it implied that these genes of WBPH might have different functions. Three WBPH *Vg* genes and the *Laodelphax striatellus* (Fallén) *Vg*-like1 gene were located on 1 clade, and 2 WBPH *VgR* genes clustered in 1 branch, which revealed that these genes had a close relationship. The study of *N. lugens* showed the similar result, which revealed that *Vg* and *Vg*-like2 were clustered together but was distant from *Vg*-like1 (Wang et al. 2021).

The putative protein of *SfVg*-like1 contains the LPD_N, DUF1943, and VWD domains, which are 3 large highly conserved domains in most insects (Tufail et al. 2010, Yao et al. 2018). The amino acid sequences of *Vg* of WBPH (Hu et al. 2019b, Zhou et al. 2020, Yi et al. 2021), *Vg* and *Vg*-like1 of *N. lugens* (Shen et al. 2019) also contains the tree domains. However, the WBPH *Vg*-like (Zhou et al. 2020) and *N. lugens Vg*-like2 (Shen et al. 2019) only contain 2 large domains of the LPD_N and VWD, and the different domains might play different roles in immune defense and nutritional supply.

The transcripts of *SfVg*-like1 expression were detected both in the nymph and adult stage (male and female), with high expression level that peaked at eclosion day 1 in male adult among the 9 detected time points. As a whole, the expression levels of *SfVg*-like1 mRNA in the nymph stage were lower than those in adult stage, and female adults had higher expression levels than male adults at day 2 and 3 (Fig. 2). The transcript expressions of *SfVg*-like and *Vg*-like1 gene in *N. lugens* also showed the higher expression levels in adult stage than those of nymph stage (Shen et al. 2019, Zhou et al. 2020). Four *Vg* genes (*ZcVg*1-4) of *Z. cucurbitae* were significantly highly expressed in female adult stage compared with those of nymph stage (Chen et al. 2022). Compared with the *SfVg*-like1 transcripts detected throughout all stages, the *SfVg* and *SfVgR* genes were both highly expressed in WBPH female adults, and their expression levels were very low in male adults and nymphs (Hu et al. 2019b, Zhou et al. 2020, Yi et al. 2021). In *N. lugens*, *Vg* transcripts were also primarily detected in female adults, whereas *Vg*-like1 transcripts were detected throughout all stages (Shen et al. 2019). The above results implied there might be the different regulatory mechanisms and physiological functions of *Vg*-like1 and *Vg* genes in WBPH and *N. lugens* development and reproduction.

RNAi technology was used to investigate the gene functions of *SfVg*-like1 in WBPH. Our results showed that *SfVg*-like1 expression levels of WBPH female adults decreased 48.5%–96.1% after injection within 1–5 days. Furthermore, the ovaries of female adults were dissected to check the silence effect of *SfVg*-like1 on their developments. For WBPH female adults within 24 h of emergence, the results of ovaries dissection at day 3 and 6 silenced with ds*SfVg*-like1 showed that knockdown of *SfVg*-like1 inhibited the ovarian developments with no or irregularly shaped immature oocytes in the ovarioles. Therefore, ds*SfVg*-like1 knockdown significantly affected WBPH ovarian development. However, the development of testes and accessory glands were not affected for male adults within 24 h of emergence injected with *dsSfVg*-like1 at day 3 and 6, although the *SfVg*-like1 expression level decreased 61.1%–90.2% after injection within 1–5 days. It revealed that *SfVg*-like1 played an essential role in the development of the WBPH female reproductive system, but had no effect on that of male adult. Previous studies revealed that knockdown of 3 WBPH *Vg* genes, MH271114 (Yi et al. 2021), MN229743 (Hu et al. 2019a), and MN296092 (Zhou et al. 2020), all can decrease the *Vg* expression levels and cause the ovaries to have less Vn in the basal oocyte and incomplete oocytes for female adults. A study on *N. lugens* showed the similar results which found that the knockdown of *Vg*-like1 in *N. lugens* female adults resulted in failure to hatch or death before eggshell emergence in 18% of offspring embryos, suggesting that it played an important role during late embryogenesis (Shen et al. 2019). However, the study of Zhou et al. (2020) found that the number of eggs laid and their hatching rate were not affected, although the expression level of *SfVg*-like was significantly downregulated after ds*SfVg*-like injection.

Many studies found that the amino acid-Target of Rapamycin (AA/TOR) and insulin pathways are involved in controlling biosynthesis and secretion of juvenile hormone (JH) and ecdysone (E) (Tufail et al. 2014, Smykal and Raikhel 2015). JH and 20-hydroxyecdysone (20E) biosynthesis pathways are essential for *Vg* synthesis (Parthasarathy et al. 2010, Gujar and Palli 2016, Santos et al. 2019). JH acts through its receptor, methoprene-tolerant (Met) (Jindra et al. 2013), and many studies found that knockdown of Met in female adults impaired the fecundity by reducing *Vg* synthesis in the fat body and Vg uptake in oocytes (Yue et al. 2018, Hu et al. 2019a). In our previous study, we found that knockdown of *SfTOR* caused the *SfVg* transcripts to decrease significantly, whereas *SfVg* silence failed to alter the expression level of *SfTOR* gene (Yi et al. 2021). JH III of American cockroach (*Periplaneta americana*) indirectly activates *Vg* expression by interfering with or inhibiting the phosphorylation of nuclear proteins bound to *Vg1*HRE (Elgendy et al. 2019). In *N. lugens*, knockdown of *NlRpn*s and *NlRac*s significantly decreased the levels of *Vg* and *Vg-*like2, but not *Vg*-like1, in the ovaries and fat bodies (Wang et al. 2021). The ds*SfVg*-like injection did not affect the *Vg* transcripts in the whole bodies of WBPH females in the study of Zhou et al. (2020). However, in this study, the transcript levels of *SfVg* and *SfTOR* were decreased significantly in female adults compared with the ds*GFP*-treated control groups after silence of *SfVg*-like1. Conversely, knockdown of *SfTOR* or *SfVg* in female adults did not affect the expression level of *SfVg*-like1.

In summary, this study identified the ORF sequence of *SfVg*-like1 and made the temporo-spatial expression analyses in WBPH different developmental stages. The results of RNAi knockdown effects revealed that *SfVg*-like1 might locate on the upstream signaling pathways of *SfTOR* and *SfVg*, and it was essential for WBPH reproduction. The regulation mechanism behind the *SfVg*-like1, *SfVg*, and *SfTOR* genes deserves further studies. All of the above results suggest that the *SfVg-*like1 gene has the potential to be a promising target for RNAi-based population management of the WBPH.

## Supplementary Material

Supplementary material is available at *Journal of Insect Science* online.

ieae013_suppl_Supplementary_Tables_S1

ieae013_suppl_Supplementary_Figures_S1

ieae013_suppl_Supplementary_Figures_S2

ieae013_suppl_Supplementary_Figures_S3
