# Supplementary figures and images for: Molecular characterization of Vitellogenin-like1 gene in Sogatella furcifera (Hemiptera: Delphacidae), and its function on reproduction
Source: J Insect Sci. 2024 Feb 27;24(1):17. doi: 10.1093/jisesa/ieae013 (PMC10898789; doi:10.1093/jisesa/ieae013)

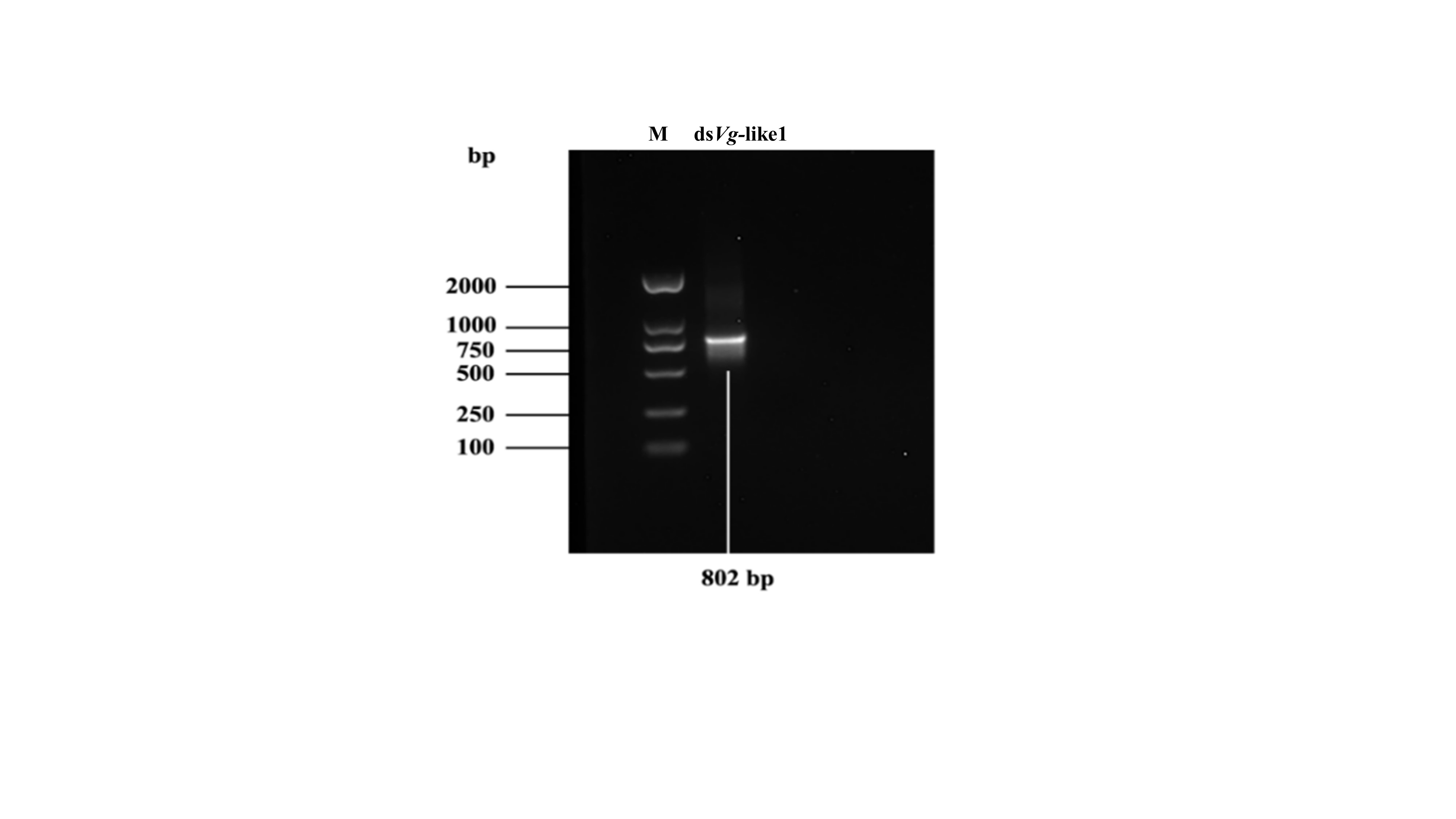

Supplement: ieae013_suppl_Supplementary_Figures_S1 [file ieae013_suppl_supplementary_figures_s1.jpeg]

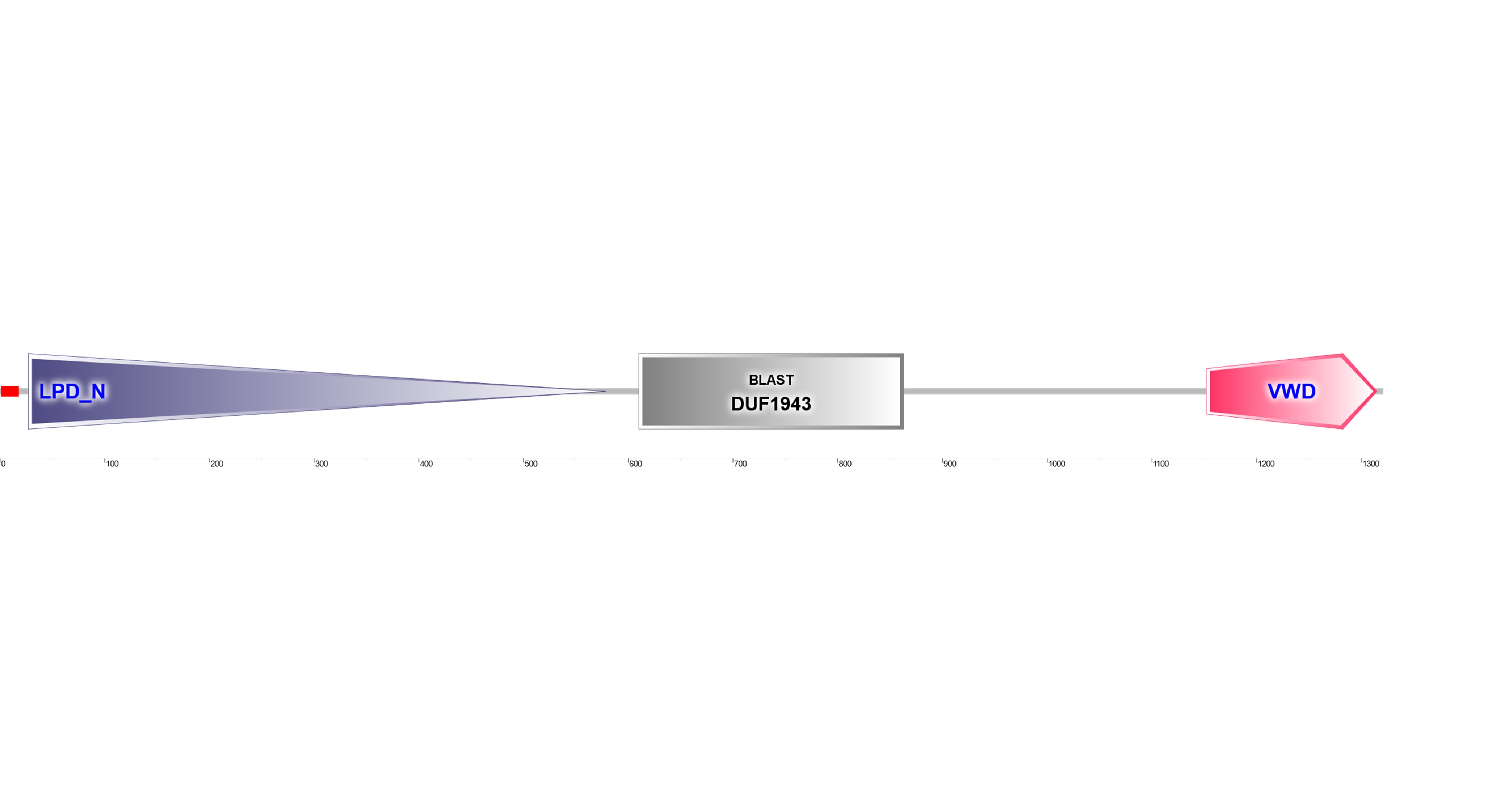

Supplement: ieae013_suppl_Supplementary_Figures_S3 [file ieae013_suppl_supplementary_figures_s3.jpeg]
