# Supplementary material for: Molecular characterization of Vitellogenin-like1 gene in Sogatella furcifera (Hemiptera: Delphacidae), and its function on reproduction
Source: J Insect Sci. 2024 Feb 27;24(1):17. doi: 10.1093/jisesa/ieae013 (PMC10898789; doi:10.1093/jisesa/ieae013)
Supplement: ieae013_suppl_Supplementary_Figures_S2 [file ieae013_suppl_supplementary_figures_s2.pdf]

1 ATGGATCGATTCGTGATAATTGTCCTAGGTCTCCTAATTGCTGGTTCAGCTTCAGAAGAAGAATATCTGTTCTTGCCAGAACATGAATCA 90
1 M D R F V I I V L G L L I A G S A S E E E Y L F L P E H E S 30

91 GTCTATGCGTGGAATGTGACTCTAACCACGGGGGCAGCTCTGCCCCAATCCGTTAGTTTATCATGGAACTTCTCGGCAAATCTCCATGTG 180
31 V Y A W N V T L T T G A A L P Q S V S L S W N F S A N L H V 60

181 CAACAGACGCCAGGCAACACTACKATTTTCAAGATAAGTGATGTGATGACCTCTGAGAACCATGCAGTTGAAACAAGACCACTGATGCAG 270
61 Q Q T P G N T T I F K I S D V M T S E N H A V E T R P L M Q 90

271 CCTTTTAAAGCAACGTTTGAGAGAGGTCTTCTGACAGGACTGGCAACAGAAGGCACGGACAAAAAATGGTCAGTCAATATGAAAAGAGCG 360
91 P F K A T F E R G L L T G L A T E G T D K K W S V N M K R A 120

361 CTCGTCACTCTTCTACAATTGGACTTGGCCAGTATGGGTCAGATTGCTTTTCTTTCGTCAGAGAGTGGAATGTACGGAACTTGTGTGACC 450
121 L V T L L Q L D L A S M G Q I A F L S S E S G M Y G T C V T 150

451 CACTATATTGTCACAAATGGCGACAAAAAGATGACAGTGCAGAAAATACTTGACATGGACTCATGCTCGAGCCTCACATCGCAAGATTCA 540
151 H Y I V T N G D K K M T V Q K I L D M D S C S S L T S Q D S 180

541 CATCAGTGGAGCAGTGCGCCAAATTTCATGTGTCCCAATAAGTATCAAAAAGAGGTAATTAGTCACAGTGAAAGGACCTACATTCTGGAT 630
181 H Q W S S A P N F M C P N K Y Q K E V I S H S E R T Y I L D 210

631 CCAAGTGCCAAGAGTCTCATCCAGTCAATCTCAAGCAAAGGAAAAATTTCACTGCATCCCTACCAGTCTCAGGCTGAAGCCCATTACTCT 720
211 P S A K S L I Q S I S S K G K I S L H P Y Q S Q A E A H Y S 240

721 CTAATAAGCCAGTCCCTGGATCTTATCAAAGTGGAAAAAGTTGATAAACTATATGAAGTGGATGAGAGCGCTGAAGAAACAAATCTCTTA 810
241 L I S Q S L D L I K V E K V D K L Y E V D E S A E E T N L L 270

811 TGGCAGCCCATCAATCTCGATCCCACCTATGGAAAACCTCCAACTAATAAGAGTGATGTTGTTCTGAAGGAGATTCAAAACATGCTGGGT 900
271 W Q P I N L D P T Y G K P P T N K S D V V L K E I Q N M L G 300

901 GAGGTGAGCGACAGCCTGTCGTGGTGGAAGGTGAAGGCGCAAGGCTTGCACAACGAAACCCTTTTCAGACTGGTCGATCTGATGTGGTGG 990
301 E V S D S L S W W K V K A Q G L H N E T L F R L V D L M W W 330

991 CTGGAGTTGGAGGACTGGCGAACACTCTACAACACTGTCACTCTGGGTACCAGCTATAGGCAGGAAACTATCCAGCAAATATTCTGGGAG 1080
331 L E L E D W R T L Y N T V T L G T S Y R Q E T I Q Q I F W E 360

1081 CTGGTGCCCGAGGTTGGTTCATCGGCGTCAACGGTGTTCGTCAAGGACTTGGTGCGCACCAGTCAGGTGAAAGGTCTGAACGCCGTCTTC 1170
361 L V P E V G S S A S T V F V K D L V R T S Q V K G L N A V F 390

1171 CTCCTCGGACGGCTGCCCTTCCGACTCCGCAATCCAACTGAGGAACTACTCTACCGGTGCGAGGACCTTTTGCGGCTGGGTGATGTAGGA 1260
391 L L G R L P F R L R N P T E E L L Y R C E D L L R L G D V G 420

1261 GAAGAAGTGAAAAACTCGGCAATTTTGTCCTTTGCTACGATGATCTATAAAACGTGTGCTGTCAGTTGCAAACCAGATACAGTCGACAGA 1350
421 E E V K N S A I L S F A T M I Y K T C A V S C K P D T V D R 450

1351 TACACTAAATTGTTCTTGGATAGATTCAAAGAAAGCACACTCCATGGAGACAGAATGTTGTATTTGCAAGCACTATGCAATATGGGATTG 1440
451 Y T K L F L D R F K E S T L H G D R M L Y L Q A L C N M G L 480

1441 CCTCAGATTCTTGACTACCTTTCACCAATTATCGCTGGTAAAACATCCAACGACCGGCATTTGAGGTTCTTGGCCACTTGGGCTGTTATA 1530
481 P Q I L D Y L S P I I A G K T S N D R H L R F L A T W A V I 510

1531 CCATCAGTGTTCACTGACCCTGGAAAGGTTCATGAGATATTCTGGCCGCTACTTGTGAACAGAACCGAGTCGCTGGAGATCAGAGTGGCT 1620
511 P S V F T D P G K V H E I F W P L L V N R T E S L E I R V A 540

1621 TCGTTAACACTGCTAATTCTGGCGAAACCCACCCCTGCCGGACTCATCAGTTTGTTCTGGTACATGGCCAATGAGCCCAGCCAACAGCTC 1710
541 S L T L L I L A K P T P A G L I S L F W Y M A N E P S Q Q L 570

1711 TATCAGTTCTACTACACCACAATACAGTCGCTCACTCAGACAACCTATCCTTGTTACACGCAACTAGGAGTAGTAGCTTCGCAGCTGGCT 1800
571 Y Q F Y Y T T I Q S L T Q T T Y P C Y T Q L G V V A S Q L A 600

1801 CGGTTCGTGCACCCGCGCTCACACAGCTGGGCGACCGGCAACTACATCCTCGACTACGAGGAGCCCGACCGCGGCTACGGAGGCCTGCTG 1890
601 R F V H P R S H S W A T G N Y I L D Y E E P D R G Y G G L L 630

1891 CAGATGCTGCTCATTGGAAGTGAGAAGACAGGGCTGCCCAATGTCATGATCATGGTGGCTGAGCAGCATGCTCTCGGCCTRACAACTGAA 1980
631 Q M L L I G S E K T G L P N V M I M V A E Q H A L G L T T E 660

1981 CATGCTGTATACCTAAAACTTGAAGGGCTGAGCGAGGCAATGAAGCACGTGGTGGGGAACAAGCCGGTCAATAGTGTAGAGAAAGTCCTC 2070
661 H A V Y L K L E G L S E A M K H V V G N K P V N S V E K V L 690

2071 GAAATGCTGCAAAACATAAAAGCACCGGACAGGGATGGCGAGAAAATCCATTTCGAATTTATTCTGAAAGCTGATGGTAGAACGGTGATG 2160
691 E M L Q N I K A P D R D G E K I H F E F I L K A D G R T V M 720

2161 ACGTATTTCATGAATGAGACCAACTTTCATAATTTGACTGCTGTTGCAAAAAAGCTGAGTTCGTTGTACTTTGAGTTTAGCGTTAACTAT 2250
721 T Y F M N E T N F H N L T A V A K K L S S L Y F E F S V N Y 750

2251 CAGCGGCTGACGTTTCCTCTGGCTCTGCTCAAGCACCAGCCGACCGATTTCGGCGTGACGGCTCTGTCTCAGGTGCGATTCTCGTCATTC 2340
751 Q R L T F P L A L L K H Q P T D F G V T A L S Q V R F S S F 780

2341 ATGTCAGCCAGGGGCCGGGTGTGCCAGGATGAGGAGGGAGCCGCCAGAAATGCTGAATTGGATTTGAGATATTCGTGGAACGGAATCACC 2430
781 M S A R G R V C Q D E E G A A R N A E L D L R Y S W N G I T 810

2431 AGTCTGAGAGTTTTCAGTCCACTGAGCAACACGTGGTATGGAGCTGATAGAAGTAGAAGTATACACATTCGAGTTCCGTTTGCCACTCAC 2520
811 S L R V F S P L S N T W Y G A D R S R S I H I R V P F A T H 840

2521 ATCACTTTGAATTTTGCCAAATCTCATCTGAAAATCGTTGCTGTCAAGCATAGAGATTTCATGGCAGGTTCACAGATGGGTGGAGTGTGG 2610
841 I T L N F A K S H L K I V A V K H R D F M A G S Q M G G V W 870

2611 CATTCTGTTACAAAATTAATTTCTGGAAGTACAACGATCGATCAACCTCACAATATAACTGATGAATGGACAATGGACTCTGAAGATTTG 2700
871 H S V T K L I S G S T T I D Q P H N I T D E W T M D S E D L 900

2701 GGAGCTAGACTTGGAGCTAGTGTCTTCGACTGTCCAGGCCAAACATTTGGAAATGCCCTACATTTACTGAAAAGAGCTTTTCTGGCGAAA 2790
901 G A R L G A S V F D C P G Q T F G N A L H L L K R A F L A K 930

2791 AATAAAAACTATCATATGCTGCCTGGTGGAGTCGCTTTGTTGGGTCTATTTTCCCTCAAAGATCAGTTGGCATTCCAACCGCCAGGGGGA 2880
931 N K N Y H M L P G G V A L L G L F S L K D Q L A F Q P P G G 960

2881 GCCTGCGGTGTTCTGTTGTCTTTTGTACCATTGTTAACACAGGTAGAACCTGTGCTACTATTGGAGAATAACCAACTAAGCCTATCAATG 2970
961 A C G V L L S F V P L L T Q V E P V L L L E N N Q L S L S M 990

2971 ACACGACGTGATGGACTTCTGTGGGAGATAAAGGCGGCAATGAAGCATCTGCATGATGGGAACAAGGAGGCCGCTTTTAAGCTGTATCAC 3060
991 T R R D G L L W E I K A A M K H L H D G N K E A A F K L Y H 1020

3061 GCGCCAAGTATTTCAGTGTCAGCTGGCGGATTCTGGAGAGTCATTCAGCTTGAGGGCGCCTTCATCATTCCGTCGAGGAAATCGGGCGTT 3150
1021 A P S I S V S A G G F W R V I Q L E G A F I I P S R K S G V 1050

3151 TTCCACCCTCCTGCTCCTATCACTGGTTATACTTTTGTCTCATGGGGCGATGCCCAGCCAAGCAATTCAGACAAGCTGTCAGTGCTTGAT 3240
1051 F H P P A P I T G Y T F V S W G D A Q P S N S D K L S V L D 1080

3241 GTGAAGGTGGTGCCAGGCAACAACATAAGCCAGCAATCTCTGTGCACTGATTTCAATCCGATTTGTCTCCAGGCATTAACTGATCTAGCT 3330
1081 V K V V P G N N I S Q Q S L C T D F N P I C L Q A L T D L A 1110

3331 GCTCGACAAACTGCCAATGTCCAGTACTTGAACCTTCCTGTTTGGTTCAAGACAGCTGCTCATGCTGTATTCCCAGAGCACTTCCAAACT 3420
1111 A R Q T A N V Q Y L N L P V W F K T A A H A V F P E H F Q T 1140

3421 GAGAGTACTTCAGCCACATTCACCTTCAACAGCCCTGTACCATTCCCATGGAACACAAAAGGTTTGTGTGCGGTTAGTAAGAGCGTAATC 3510
1141 E S T S A T F T F N S P V P F P W N T K G L C A V S K S V I 1170

3511 CTGACTTTCGACAATGCAACTCTAGCCTCTTCGATGCCTGAAGAATACACCCTCGCTGTAGCCGACTGTTCAGCTCATAAACAATTCGCC 3600
1171 L T F D N A T L A S S M P E E Y T L A V A D C S A H K Q F A 1200

3601 ATTCTCACAAAAAAAGTACCCGAGAGTGACACTCTGGTAGTGAGACTCCTGTTTGGAACAAAAGACGTTGAAATAATCCCGACATTAGGT 3690
1201 I L T K K V P E S D T L V V R L L F G T K D V E I I P T L G 1230

3691 GGCAAACTGAAGTTTGTTCTCAACGGAATACCACTCTCAGACACTGAGTTCACACAAAACTTTGGAAACTCTTCAACCACAGATATGCTA 3780
1231 G K L K F V L N G I P L S D T E F T Q N F G N S S T T D M L 1260

3781 ATCTCACTAAAGGACAATGGCATGACAGAAGCTGAGTTGGAGAACGGGGTAGTTCTCCAGCACTATAACTCCACAATAGTTGTTCTTGTA 3870
1261 I S L K D N G M T E A E L E N G V V L Q H Y N S T I V V L V 1290

3871 CCGGGGCTGTTCCGCAGCTTCACGTGCGGATTATGTGGAGACTTCAATAGTGACACCAACAACGACCCCATACACATGTTCACCTACATC 3960
1291 P G L F R S F T C G L C G D F N S D T N N D P I H M F T Y I 1320

3961 AAATGA
1321 K *

Notes: ATG is the start codon and TGA is the stop codon. The underlined section is the signal peptide sequence.
